# Master Proteins Dictate Retinal Differentiation Timetable

**DOI:** 10.1371/journal.pbio.0040293

**Published:** 2006-08-15

**Authors:** Liza Gross

The embryonic construction of the vertebrate retina is a highly ordered affair. Following a precise timetable, six different specialized cell types emerge from a mass of identical, proliferating cells. The process of retinal cell differentiation, when so-called progenitor cells stop dividing and choose among the six fates, depends primarily on homeobox genes, major regulators of embryonic patterning. How these genes control the timing of retinal cell differentiation has remained an open question—until now.

In a new study, Sarah Decembrini, Federico Cremisi, and colleagues show that three homeobox genes work in conjunction with a cellular timepiece that determines the sequential emergence of distinct cell types. Surprisingly, the schedule of both homeobox gene expression and retinal cell differentiation is controlled by the translation, rather than by the transcription, of the genes.

Retinal cells transform light signals into visual information for further processing in the brain. After light stimulates the rod and cone photoreceptors, visual signals travel to horizontal and bipolar cells, which in turn interface with amacrine cells. Ganglion cells, which then relay these signals to the brain, are the first-born cells—that is, the first to exit the cell cycle and stop dividing. Though their birthdays vary somewhat by species, the horizontal, cone, and amacrine cells come next, then the rod and bipolar cells.

Decembrini et al. suspected that cell identity may be tied to cell cycle progression because different retinal cell types are produced when cell cycle length is manipulated. To test this hypothesis, they studied a subset of homeobox genes, including *otx5*, which supports photoreceptor differentiation, and *vsx1* and *otx2*, which promote bipolar differentiation. Working with *Xenopus* frogs, a classic developmental biology model, they found that each of the homeobox genes was expressed in sequence, in different cells. By mid-stage retinal development (stage 34), the messenger RNA (mRNA) transcripts of all three genes were expressed, but only Xotx5 proteins were detected. Xvsx1 and Xotx2 were detected at stages 37 and 38-39, respectively. By stage 42, Xotx2 and Xvsx1 proteins were observed in bipolar cells, while Xotx5b was detected only in photoreceptors. These results indicated that the genes had been regulated after transcription and were expressed as proteins after cells exited the cell cycle.

What controlled the genes’ translation into protein? To find out, the researchers linked a specific sequence of each homeobox gene—called the three prime untranslated region (3' UTR)—with the gene encoding green fluorescent protein (GFP). These GFP sensors indicated how the 3' UTR affects expression of the gene. They delivered the DNA of sensors into embryos at an early stage of retinal development (stage 17-18), using a technique called lipofection. GFP proteins were detected only in photoreceptors (the *Xotx5b* sensor) and bipolar cells (*Xvsx1* and *Xotx2* sensors). Thus, the 3' UTRs of these genes had blocked GFP translation into protein in all but late-developing retinal cells. The 3' UTRs were able to do this because they contain sequences (called cis-regulatory sequences) that can interact with microRNAs—a class of gene-repressing RNAs that bind to complementary sequences of RNA and mediate mRNA destruction. (Future experiments must confirm whether these sequences do in fact interact.) The GFP sensors were detected at the same stages as their corresponding homeobox proteins had been in the previous experiments. This timing, it turned out, coincided with the birthdates of the photoreceptors and bipolar cells.

**Figure:**
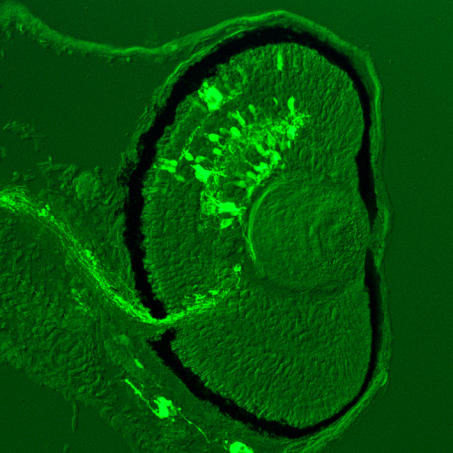
Green fluorescent protein traces different types of lipofected cells in the neural retina of a *Xenopus* tadpole, some of which (ganglion cells) generate fibers of the optic nerve

The correlation between the timing of protein expression and the photoreceptor and bipolar cell birthdates prompted the researchers to examine the effect of cell cycle progression on protein translation. By blocking cell cycle progression with drugs that inhibit DNA replication, they found that *Xotx5b*, *Xvsx1*, and *Xotr2* require progressively longer cell cycles for efficient translation. And the attenuated production of Xotx5b and Xvsx1 proteins after cell cycle inhibition, they found, reduced the number of photoreceptor and bipolar cells—an effect that was reversed when the proteins were overexpressed, supporting the connection between protein expression and cell identity.

Altogether, these results indicate that a post-transcriptional mechanism regulates when these proteins are expressed and in which cells. This mechanism operates in synch with a cellular clock that measures cell cycle length to generate the later developing photoreceptors and bipolar cells. The next step will be to determine how these findings apply to other genes controlling retinal cell fate, and then to identify the molecular mechanisms driving translational inhibition.

